# Synthesis, Characterization, and Electrochemical Evaluation of Electrodeposited NiCuZn Powders for Urea Oxidation

**DOI:** 10.3390/ma19101973

**Published:** 2026-05-10

**Authors:** Agata Kołkowska, Wojciech Lisieński, Łukasz Gardas, Weizhi Shang, Aleksander Gąsior, Artur Maciej, Marta Wala-Kapica, Wojciech Simka

**Affiliations:** 1 Faculty of Chemistry, Silesian University of Technology, Strzody St. 9, 44-100 Gliwice, Poland; agata.kolkowska@polsl.pl (A.K.); wl301143@student.polsl.pl (W.L.); lg304926@student.polsl.pl (Ł.G.); aleksander.gasior123@gmail.com (A.G.); artur.maciej@polsl.pl (A.M.); marta.wala-kapica@polsl.pl (M.W.-K.); 2 Chemistry Students Research Society, Faculty of Chemistry, Silesian University of Technology, 44-100 Gliwice, Poland; shangwz@stumail.ysu.edu.cn; 3 Yanshan University, 438 Hebei Ave West Section, Haigang District, Qinhuangdao 066000, China; 4 Department of Chemistry, Faculty of Medicine and Life Sciences, University of Latvia, LV-1004 Riga, Latvia

**Keywords:** urea oxidation reaction (UOR), direct urea fuel cell (DUFC), catalytic oxidation of urea, powder, nickel-based catalyst

## Abstract

The growing demand for sustainable energy technologies has intensified interest in direct urea fuel cells as an environmentally friendly energy conversion system. In this work, a ternary NiCuZn electrocatalyst is synthesized via a single-step electrodeposition process, offering a rapid and scalable alternative to commonly used hydrothermal or multistep fabrication routes. Structural and compositional analyses (SEM, EDX) confirm the formation of coral-shaped particles of NiCuZn powders. Electrochemical evaluation in alkaline media demonstrates that powders of both tested variants exhibit clear anodic activity, with peak potentials in the range of 0.4–0.6 V_vs Ag|AgCl (sat. KCl)_. Zinc presence was confirmed also after the process. Upon urea addition, a pronounced enhancement in anodic current density is observed. Notably, variant NiCuZn powder, which was produced using higher current density during electrodeposition, shows superior catalytic activity from approximately 0.4 V_vs Ag|AgCl (sat. KCl)_, reaching a maximum of 10 mA/cm^2^ near 0.75 V_vs Ag|AgCl (sat. KCl)_, and stability, which are attributed to its highly homogeneous microstructure and dynamic surface activation mechanism uniquely by partial zinc leaching during operation. These findings demonstrate that electrodeposited NiCuZn systems can deliver competitive performance despite their structural simplicity, highlighting their potential as cost-effective and scalable anode materials for direct urea fuel cell applications. We address a critical bottleneck in fuel cell manufacturing by replacing time-intensive hydrothermal syntheses with a rapid, highly scalable electrodeposition method. Furthermore, the identification of zinc-leaching mechanisms provides crucial new insights into dynamic catalyst activation, moving beyond traditional, static anode designs.

## 1. Introduction

The constant increase in demand for electricity results from the development of civilization and the constantly growing human population. Intensive exploitation of fossil fuel deposits, i.e., coal, oil, and natural gas, and the conventional way of electricity generation, has a significant environmental impact, leading to climate change, depletion of natural resources, and environmental degradation [1]. Additionally, political tensions in fossil fuel-rich parts of the world can cause worldwide market turbulence, as it did during the oil crisis in 1973 and during the recent Strait of Hormuz obstruction [2,3].

Current research increasingly focuses on sustainable development and the search for alternative energy sources [4]. In addition to the well-known renewable energy sources, the energy sector related to the use of fuel cells is developing very intensively [5,6,7,8,9]. They would be a good alternative, especially for the automotive industry, due to limitations associated with defects of electric cars and the difficulties associated with the lifetime and recycling of lithium-ion batteries, especially considering the European Union‘s suppression of the sale of vehicles with engines emitting CO_2_ by 2035 [10].

A fuel cell is a device that converts the chemical energy of the fuel into electrical energy through a chemical reaction within it. This enables the avoidance of efficiency losses associated with the conversion of thermal energy into kinetic energy, unlike conventional electricity generation methods. To function, a fuel cell requires fuel and at least two electrodes, an anode and a cathode, at which redox reactions take place. The most well-known fuel used in cells is hydrogen [5,6,11,12], with the growing popularity of liquid fuels like simple alcohols, i.e., methanol [12,13,14] or ethanol [15,16].

Additionally, the usage of liquid fuels is possible using the framework of the existing fuel infrastructure, which would simplify the transition between fossil fuels and renewable energy [17]. One of the more interesting compounds which can serve the role of fuel in a direct fuel cell is urea. Due to its abundance, low costs, biodegradability, stability, and safety, urea is a subject of increasing interest. As a product of mammal metabolism, it is present in agricultural and civil wastewater. Additionally in environment it is oxidized by the urease—an enzyme catalyzing the reaction of urea oxidation to ammonia and carbon dioxide—which means that usage of urea as a fuel will not increase the total CO_2_ emissions [6,7,12,17,18,19,20,21].

Appropriate materials are required to promote efficient fuel oxidation and allow for maximum acquisition of electrical energy on the cell, while being durable and maintaining their activity over time [7,22]. Due to the limited availability of anode materials or the still insufficient activity and efficiency, the issue of catalytic materials is currently one of the major research topics in the direct fuel cell sector.

One of the materials that can be used in the catalytic oxidation of urea is nickel, which in nature serves as the active-site metal centre of the urease [18,20,23]. Noble metal-based catalysts generally show limited activity toward the urea oxidation reaction, while Ni-based materials are considered the most effective catalysts due to their ability to form active NiOOH species in alkaline environment [21,24,25,26,27]. Despite low costs and high availability, nickel itself has insufficient activity for use in a fuel cell, which is why efforts are underway to increase it [9,19,28,29]. The activity of nickel-based electrocatalysts can be increased by adding other metals, like iron, copper, cobalt, or zinc, to increase the activity of the system. Additionally, they often contain graphene or graphene oxide in their structure that promotes facile charge transfer and enhanced material dispersion [18,19,30,31,32,33].

Copper and zinc, among other dopants, are the most noteworthy. Their integration into nickel-based systems emerged as an effective strategy to increase their synergic effect. Copper modifies the electronic structure of the nickel centre, lowering the onset potential required for the formation of the active NiOOH. It also facilitates the cleaving of bonds present in the urea molecule. Zinc modulates the d-band centre, which promotes the creation of a porous, defect-rich structure of the fuel cell, required to increase its efficiency. Moreover, the increased area increases the amount of possible adsorption sites [9,13,29,34,35]. As for the form of the anode material, there are materials in the form of nickel sponges, powders, or ordinary electrodes [7,18,34,36]. Nanomaterials and advanced metal–organic structures are also used, such as carbon nanosheets or nanostructured NiO@C [7,12,17,19,23,37]. The structure of such anode materials also influences fuel cell efficiency through the size of the contact area between the catalyst and the fuel, which increases with the growth of the electrochemically active surface area [12,20,23,34,38].

Another difficulty encountered when working with fuel cells is the way they are manufactured, since for a mass scale production they should be cheap and available [17,36]. One of the important obstacles in this process is obtaining anode material, since currently, most of such materials are obtained by expensive and time-consuming hydrothermal methods [5,19,34]. Alternative method could be to produce catalytic materials using the electrodeposition process [22,39]. It provides greater control over the process and final state, while reducing production costs in a shorter time [30]. One of the greatest advantages of the materials synthesis by electrodeposition is that the flexibility of this method allows the amount of synthesized material to be easily scaled by adjusting electrode size, without affecting material properties, and thus can be easily adjusted to the market’s demand, which is crucial in a fast-paced economy, following the client’s demand. Electrodeposition can be used for cathodic electroreduction and for preparation of composite or metallic coatings, while anodic electrodeposition can be used for synthesis of oxides. Properties of the obtained materials are directly related to the process parameters, such as current density, and an increase in electrode size does not inherently affect the intrinsic properties of the deposited materials [40,41,42].

Beyond operational simplicity and reduced synthesis time, the single-step electrodeposition route provides distinct structural advantages over traditional hydrothermal methods. By applying high current densities, the synthesis leads to the dynamic hydrogen bubble template (DHBT) mechanism. The deposition process uses the constantly created hydrogen bubbles as templates, generating a hierarchical, highly porous, coral-like 3D architecture. Consequently, this method structurally engineers a catalyst with an inherently maximized contact area for the electrolyte, without the need for external templating agents, binders, or further processing [43,44].

While many Ni-based ternary systems rely on static electronic synergistic effects, NiCuZn catalyst proposed in this study introduces a dynamic surface activation pathway. As demonstrated by our post-operative compositional analysis, partial zinc leaching occurs during the urea oxidation reaction (UOR). This process acts as a continuous surface-modifying mechanism that exposes fresh nickel adsorption sites and increases surface area gradually, which distinguishes described systems from conventional, stable alloys—by transforming the degradation of an electrode into a functional catalytic advantage [45,46,47].

In this study, a ternary NiCuZn catalyst was synthesized via a single-step electrodeposition method and evaluated toward the urea oxidation reaction in alkaline media. The novelty of this work lies in introducing a NiCuZn system as a catalyst for urea oxidation, which remains largely unexplored among Ni-based materials. In addition, the use of earth-abundant and low-cost elements, combined with a simple and scalable electrodeposition route, provides a practical advantage over commonly used multi-step synthesis methods. This aspect is particularly relevant in the context of global supply chain instability and geopolitical tensions affecting the availability of critical raw materials. Importantly, this approach enables direct control over catalyst composition and properties through electrochemical parameters, without modification of the solution used in the electrodeposition process. Furthermore, the observed compositional changes during operation (e.g., partial zinc leaching) suggest that surface evolution may play an important role in catalytic performance and should be considered in catalyst design. The obtained powders were comprehensively characterized in terms of morphology and elemental composition. Furthermore, electrochemical investigations, including assessment of their activity and stability towards the urea oxidation reaction (UOR), and durability in alkaline media.

## 2. Materials and Methods

### 2.1. Material Synthesis

The chemicals used for electrodeposition (copper sulfate, nickel sulfate, zinc sulfate, ammonia, and citric acid) were of p.a. grade, used without further purification, and purchased from Chempur (Piekary Śląskie, Poland).

The electrodeposition process was conducted as described previously. Briefly, the electrodeposition was carried out in an electrolyzer equipped with a mechanical stirrer and two nickel anodes. A copper plate, onto which the synthesized powder was deposited, was used as the cathode. Before electrodeposition, pure copper sheets were polished with SiC sandpaper (grade 800, then 1000), washed with distilled water, and degreased in isopropanol using ultrasound for 5 min. Immediately before the electrodeposition process, samples were immersed in 40% nitric acid solution for 1 min, washed with distilled water, and immediately inserted into the electrolyte to prevent copper oxidation in air [48].

The electrolytic bath was prepared according to Table 1, by dissolving the ingredients in water; the pH of the resulting solution was then adjusted to 9 using 25% ammonia solution.

The electrodeposition process was carried out at current densities of 15 and 20 A dm^−2^ for 10 and 5 min, respectively, to minimize powder loss. After the electrodeposition process, the deposited material was removed from the copper plates using ultrasounds, collected as powder, and used for further investigations. Sample labels were related to the current density used in text samples, which were called NiCuZn15 and NiCuZn20.

### 2.2. Morphology and Chemical Analysis

Morphology of the surface was studied using a Scanning Electron Microscope (SEM), Phenom ProX (ThermoFisher, Eindhoven, The Netherlands), at an accelerating voltage of 15 kV. Energy-dispersive X-ray spectroscopy (EDX) was performed using the same instrument.

#### 2.2.1. Electrochemical Measurements

Electrochemical measurements were carried out using a three-electrode configuration. An Ag|AgCl electrode filled with saturated KCl solution served as the reference electrode, while a glassy carbon rod was used as the counter electrode. All potentials reported in this study were measured with the same electrode setup and are expressed *versus* Ag|AgCl_(sat. KCl)_.

The working electrode was prepared by drop-casting 5 μL of catalytic ink, containing prepared NiCuZn powder, on the surface of a polished glassy carbon electrode. Before each measurement, the electrode was polished using aluminum oxide paste (of 3 μm diameter), washed with distilled water, and dried.

Catalytic ink was prepared by mixing 20 μg of NiCuZn powder and 60 μL of commercial Nafion^®^ copolymer produced by DuPont (Wilmington, DE, USA) and isopropanol solution in a 1:10 ratio. The suspension was homogenized using ultrasounds until deep, black ink was obtained. Immediately after homogenisation, the suspension was drop-casted onto the centre of a glassy carbon cleaned earlier. Samples were then dried overnight at room temperature.

#### 2.2.2. Electrochemical Activity Measurements

Cyclic voltammetry (CV) measurements were conducted in 0.5, 1, and 2 M KOH solutions. Measurements were performed in a range from 0 to 0.75 V_vs Ag|AgCl (sat. KCl)_ with a scan speed of 0.01 Vs^−1^. For further analysis, the best performing solution was chosen, with the highest stability and peak potential, which was observed in 1 M KOH solution. The measurements were performed on three independent samples, and the average and standard deviations were calculated.

#### 2.2.3. Electrocatalytic Urea Oxidation

Samples’ activity towards urea oxidation was measured using cyclic voltammetry. Measurements were conducted for chosen samples in a solution of 1 M KOH and 0.15 M urea in 0 to 0.75 V_vs Ag|AgCl (sat. KCl)_ with a scan speed of 0.01 Vs^−1^. The urea concentration corresponds to approximately half of the typical concentration found in human urine.

#### 2.2.4. Stability Measurements—Chronoamperometry

Stability of the samples was measured using chronoamperometric methods, performed in a solution of 1 M KOH and 0.15 M urea. Measurements were conducted by polarization to the anodic peak potential, observed during cyclic voltammetry scans for 30 min. The measurements were performed on three independent samples, and the average and standard deviations were calculated.

#### 2.2.5. Stability Measurements—Multicycles

In total, 20 consecutive CV scans were performed to test the stability of the samples. The measurements were performed on three independent samples, and standard deviations were calculated and included in the figure to reflect the reproducibility and variability of the results. Each cycle was performed by applying potential from 0 to 0.75 V_vs Ag|AgCl (sat. KCl)_ with a scan speed of 0.01 Vs^−1^. Measurements were performed in a solution of 1 M KOH and 0.15 M urea.

## 3. Results

### 3.1. SEM and EDX

**Figure 1:**
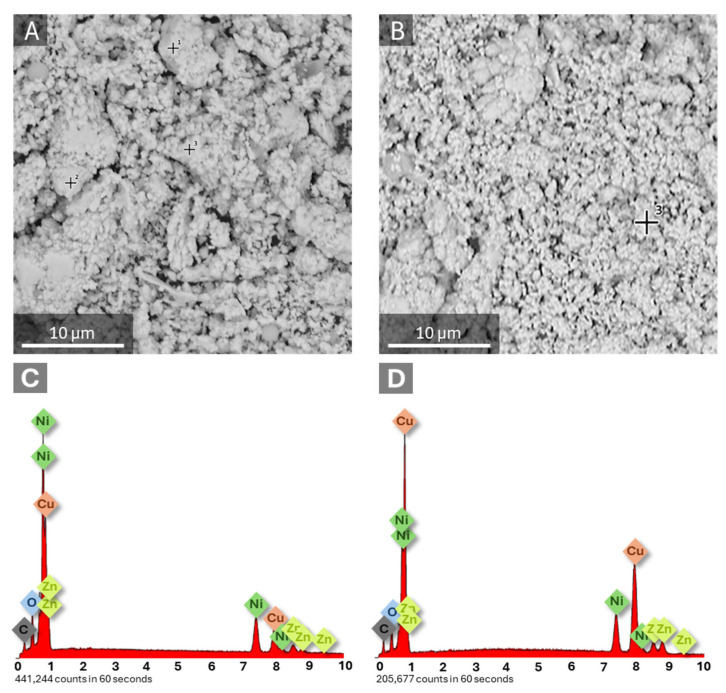
Representative SEM images and EDX spectra of NiCuZn powders synthesized at different current densities as NiCuZn15 (**A**,**C**) and NiCuZn20 (**B**,**D**), showing morphology differences and elemental composition. On the SEM images are placed markers where EDX analysis were taken.

To analyze the homogeneity and grain size of the powders and compare the two investigated variants in this study in terms of both their physical characteristics and chemical composition, SEM imaging and EDX analysis were performed (Figure 1).

The morphology of the prepared NiCuZn powders is presented in Figure 1, where noticeable differences in surface structure and particle agglomeration can be observed between the two samples synthesized with varying current density. In both cases, the materials exhibit a rough, granular structure composed of interconnected and densely packed coral-shaped particles. Similar hydrogen-bubble-assisted morphologies have been reported for electrodeposited nickel-based materials in our previous work for NiCuGO for methanol oxidation and Ni-Cu-Fe for urea oxidation [30,43,44,48]. This morphology is characteristic of materials prepared using the dynamic hydrogen bubble template (DHBT) mechanism, which occurs during electrodeposition at high current densities, where evolving hydrogen bubbles act as a transient template, generating a porous and highly developed surface structure [43,44]. The different particle sizes are related to the size of hydrogen bubbles formed during deposition. The use of higher current density leads to the formation of a larger number of smaller hydrogen bubbles, rapidly forming and detaching from the cathode surface. As this process is becoming more dynamic the final material is of smaller size, but more branched particles [43].

The NiCuZn15 powder (Figure 1A) displays a more heterogeneous morphology than the NiCuZn20 sample. The NiCuZn20 sample (Figure 1B) shows a more uniform and finer granular structure, with particles more evenly distributed and smaller in size compared to NiCuZn15. In addition, the observed morphology for both samples exhibits features resembling dendritic structures, which may contribute to the development of a highly developed surface area.

**Figure 2:**
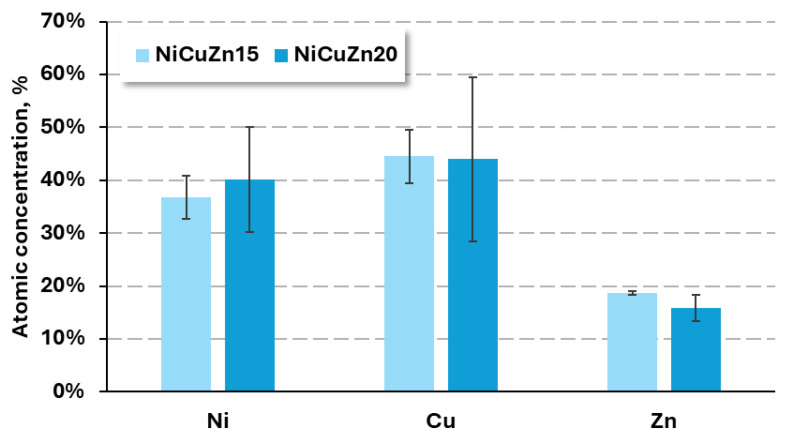
Atomic concentrations of Ni, Cu, and Zn in NiCuZn powders (NiCuZn15 and NiCuZn20) determined from EDX spectra.

EDX spectra reveal the presence of nickel, copper, and zinc in the examined samples, along with carbon and oxygen, which originate from the carbon tape support or from ambient air contamination. Figure 2 presents the atomic concentrations of the key components- nickel, copper, and zinc. The relative proportions of the elements remain broadly similar across the two formulations, with Cu being the most abundant element in both samples, followed by Ni and Zn.

Specifically, the NiCuZn15 sample exhibits approximately 37 ± 4% nickel, 45 ± 5% copper, and 18 ± 1% zinc (atomic %). The NiCuZn20 powder shows slightly higher nickel content, which is around 40 ± 10% and 44 ± 15% copper. The zinc content is lower, remaining roughly constant at about 16 ± 2%.

The lower current density applied during the synthesis of the NiCuZn15 powder resulted in a reduced nickel content and a slightly higher zinc contribution compared to NiCuZn20. However, the overall elemental compositions remained similar, and the change in current density did not lead to significant differences in the atomic percentages of the constituent elements.

### 3.2. Electrochemical Tests

#### 3.2.1. KOH Concentration Influence

The initial stage of the electrochemical activity assessment involved evaluating the influence of the KOH concentration on the performance of the proposed electrocatalyst. KOH solutions with concentrations of 0.5, 1, and 2 M were used.

**Figure 3:**
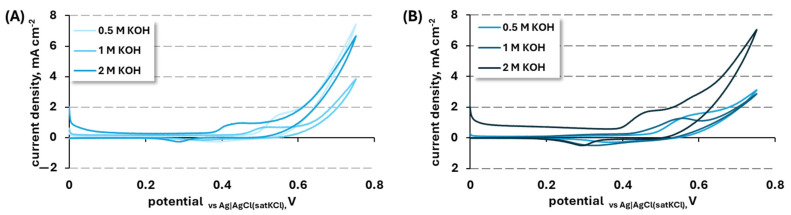
Representative CV curves recorded for NiCuZn15 (**A**) and NiCuZn20 (**B**) powders in different concentrations of KOH (0.5 M, 1 M, and 2 M).

The results of cyclic voltammetry of NiCuZn15 and NiCuZn20 in various potassium hydroxide concentrations are presented in Figure 3. Both samples display an anodic peak during the forward CV scan, at approximately 0.45–0.55 V_vs Ag|AgCl (sat. KCl)_. The observed peak is related to the oxidation of nickel from the Ni(II) to the Ni(III) state, where in an alkaline environment nickel hydroxide is oxidized into nickel oxyhydroxide as shown in Equation (1). This reaction is crucial for the electrocatalytic activity of proposed materials, since NiOOH is the active agent for the oxidation of simple organic molecules, including urea. The presence of such anodic peaks in the reported potential window is a promising sign for the electroactivity of the proposed system towards UOR. During the reverse scan, a cathodic peak is observed, related to the reverse reaction of nickel oxyhydroxide reduction back to nickel hydroxide [28,40,49].

(1) NiOH2+OH−   N←  reverse scan  →forward scaniOOH+H2 

Metallic dopants, such as Cu and Zn, can increase the activity of the Ni-based electrocatalytic system due to the d-band centre modulation. Their influence is also visible as a result of easier adsorption of reactants and desorption of products, leading to increased activity while preventing poisoning from the catalyst [50,51,52].

Additionally, similar to nickel, copper can also form its oxyhydroxide in the alkaline media after its polarization to the proper potential and can play the role of electrocatalyst for UOR [53,54].

Unlike copper, in reaction conditions, zinc is most probably partially dissolving, leading to increased material activity due to the increased amount of reaction active centres in contact with the solution. Another possibility is the formation of a zinc oxide layer, which would lower the electrode activity by blocking the electrode surface. Such phenomena would explain why the process taking place resulted in obtaining the results with a higher standard deviation, as higher OH^-^ ion concentrations are leading to the formation of a thicker ZnO layer, leading to locally lower activity and, as a result, lower repeatability of the results [55,56].

**Figure 4:**
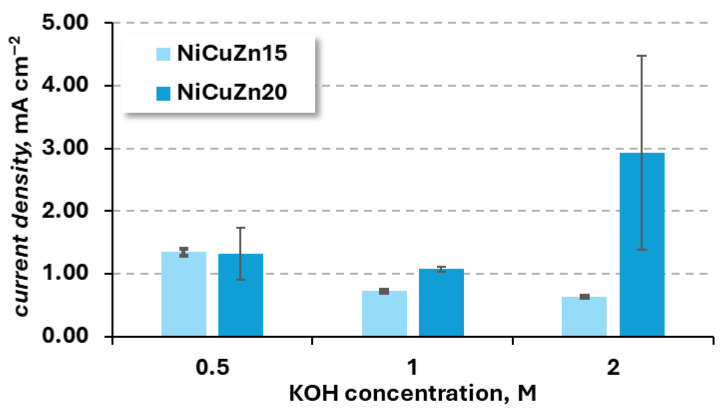
Influence of KOH concentrations (0.5 M, 1 M, and 2 M) on the anodic peak current density for NiCuZn15 and NiCuZn20 powders.

**Figure 5:**
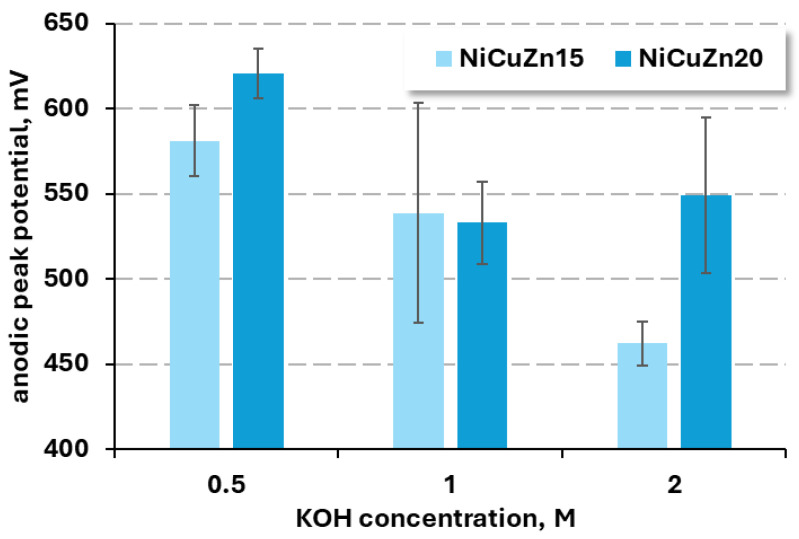
Influence of KOH concentrations (0.5 M, 1 M, and 2 M) on the observed anodic peak potential (*E*_pA_) for NiCuZn15 and NiCuZn20 powders.

To improve the clarity of the results, the anodic peak current densities and corresponding potentials are presented in bar charts (Figure 4 and Figure 5).

The anodic peak current density observed for the NiCuZn20 sample in 1 and 2 M KOH is higher than that of NiCuZn15, which suggests that this modification should be characterized by higher electroactivity towards urea oxidation. In 0.5 M KOH, both variants show similar anodic peak current density, and both materials show the lowest current response at 1 M KOH. However, the dependence of anodic peak current density used in the production of catalysts on electrolyte concentration exhibited different trends for the two catalysts. These differences can likely be attributed to variations in the catalyst structure, particularly to differences in the extent of surface development, leading to differences in their electrochemically active areas. This property of the material can be examined using electrochemical methods; however, in this paper, the strategy of reporting the material activity in regard to its mass (specific activity) was chosen. The practical implementation of material electroactivity in terms of its mass have prevailed, despite clear advantages of electrochemically active surface (ECSA) examination.

As presented in Table 2 for sample NiCuZn15, the highest anodic peak current (*i*_pA_) was observed in the most diluted solution, while increasing the KOH concentration led to a lowering of the average peak current value. The activity of OH^-^ ions strongly influences the NiOOH formation (Equation (1)), which is why it is expected an increased *i*_pA_ in more concentrated solutions would be observed. With increasing concentrations of OH^-^ ions, the conductivity of the electrolyte improves, and the presence of a higher amount of hydroxide ions near the electrode surface facilitates its transport to the catalyst active centres via diffusion. Lowering of the observed *i*_pA_ with increasing KOH concentration might be related to the fact that, in 0.5 M, all of the reaction active centres have taken part in the reaction, and further increases in KOH concentration have caused the OH^-^ diffusion to be slower, i.e., due to the higher viscosity of the solution [23,29,34,57]. In contrast, for sample NiCuZn20, the highest *i*_pA_ was observed in 2 M KOH solution but with significant standard deviation, which was probably related to the stability of the sample in reaction conditions. The second-best result for this modification was observed in the most diluted solution, and the lowest average peak current density was registered for 1 M KOH. However, when we consider the standard deviations of the results from 0.5 M and 1 M KOH solutions, the activity of the sample of NiCuZn20 is similar, with a better repeatability observed during experiments in 1 M KOH solution. Similarly, as in the case of the NiCuZn15 catalyst, the reaction rate of NiCuZn20 oxidation seems to be controlled by the OH^-^ ion diffusion. A lower standard deviation observed in the case of measurements conducted in 1 M KOH solution might be related to the fact that this modification could be characterized by higher stability than NiCuZn15, but such a conclusion cannot be confirmed without further studies of the reaction mechanism.

Lowering of the peak current density with increasing concentrations of potassium hydroxide might be caused by a lack of system stability in the given conditions. Dissolution of the formed oxyhydroxides can lead to decreasing of the rate of the anodic process, which is observed as a lowering of the registered peak current density [58]. The presence of Zn in the system, which during the contact with air can form the oxide on its surface, might lead to formation of amphoteric Zn(OH)_2_ [59,60]. Its dissolution in an alkaline environment, alongside NiOOH dissolution, might be a cause for such phenomena, but its confirmation would require further investigation. Lowering of the peak current density with increasing concentrations of potassium hydroxide might be caused by a lack of system stability in the given conditions. Dissolution of formed oxyhydroxides can lead to decreasing of the rate of the anodic process, which is observed as a lowering of the registered peak current density [58]. The presence of Zn in the system, which during the contact with air can form the oxide on its surface, might lead to formation of amphoteric Zn(OH)_2_ [59,60]. Its dissolution in an alkaline environment, alongside NiOOH dissolution, might be a cause for such phenomena, but its confirmation would require further investigation.

For both proposed modifications, the lowest standard deviation was observed in a 1 M KOH solution. Despite higher average values observed in more and less concentrated electrolytes, the standard deviations of the average *i*_pA_ were significant enough to lead to the choice of 1 M KOH for the supporting electrolyte for UOR studies.

The effect of KOH concentration on the anodic peak potential of NiCuZn15 and NiCuZn20 powders is shown in Figure 5. Both materials exhibited distinct trends in response to varying hydroxide ion concentrations. For NiCuZn15, the lowest *E*_pA_ was observed in 2 M KOH. In contrast, NiCuZn20 showed its minimum anodic peak potential at 1 M KOH, while the value increased at both lower and higher concentrations. Despite these differences in performance trends, the smallest standard deviations for both materials were recorded at 0.5 M KOH.

For further research, 1 M KOH was chosen because of the compromise between repeatability (low standard deviation) of observed anodic peak current density and low anodic peak potential, meaning that the reaction required less energy input to occur.

Of the two investigated variants, NiCuZn20 exhibited superior activity, which can most likely be correlated with its surface morphology, particularly its finer structural features. The anodic peak current density was higher than that of NiCuZn15; therefore, subsequent investigations were conducted exclusively using this sample.

#### 3.2.2. Urea Oxidation

**Figure 6:**
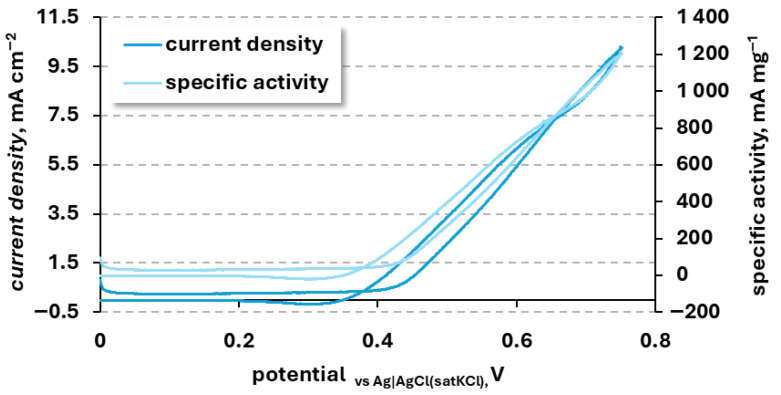
Representative CV scan recorded for NiCuZn20 powder in 1 M KOH solution containing 0.15 M urea.

Figure 6 presents the cyclic voltammogram of the NiCuZn20 powder recorded in 1 M KOH with the addition of 0.15 M urea, corresponding to approximately half the typical urea concentration found in human urine. Specific activity represents the current relative to the mass of a catalyst. A distinct anodic current increase is observed starting around 0.4 V_vs Ag|AgCl (sat. KCl)_, followed by a sharp rise at higher potentials, reaching a maximum current density of 8.69 ± 3.13 mA cm^−2^ (1022 ± 368 mA mg^−1^) near 0.75 V_vs Ag|AgCl (sat. KCl)_. During the measurements, urea decomposition occurs, leading to the formation of products (Equation (2)) [28,61].

CO(NH_2_)_2_ + NiOOH + H_2_O → Ni(OH)_2_ + products (2)

It has been reported in the literature that these products, particularly CO, may adversely affect the efficiency of the electrocatalyst, as they can induce catalyst poisoning. However, the presence of OH^-^ ions facilitates the removal of poisoning intermediates from the catalyst surface, thereby restoring and enhancing its catalytic activity [28,31].

The onset potentials, peak potentials, and current densities for nickel-based catalysts in anodic urea oxidation are listed in Table 3.

Table 3 compiles the literature’s data on selected nickel and nickel-based metallic materials employed as anodes for the electrochemical oxidation of urea in alkaline media. It is crucial to account for the disparity in testing conditions. Many high-performing catalysts reported in the literature were evaluated at higher urea concentrations (0.33 M or higher) and varying alkaline concentrations, which naturally drive higher current densities. By standardizing the potentials to the RHE scale, we provide a more accurate baseline.

Even at a lower urea concentration, the onset potential of NiCuZn remains competitive with complex architectures. The literature clearly indicates that the presence of nickel alone is insufficient to ensure high catalytic activity. In contrast, modifying nickel through alloying with other metals or incorporating hybrid structures effectively lowers the reaction potential and enhances current density, which is advantageous for urea oxidation.

Relative to conventional nickel-based materials such as NiO, the NiCuZn powders investigated in this study exhibit electrochemical activity comparable to that of other multicomponent catalysts synthesized via more elaborate routes. In particular, NiCuZn20 shows a pronounced increase in anodic current in the presence of urea, with an onset potential of approximately 1.42 V_vs RHE_ (0.4 V_vs Ag|AgCl (sat. KCl)_). This value lies well within the range reported for Ni-based materials modified with transition metals, including Ni-Zn-Co, NiCuFe, Ni@C, and Ni/Gr systems [9,17,18,30].

Comparing the NiCu-based materials prepared by electrodeposition from an aqueous solution, which were NiCuGO20, NiCuFe, and NiCuZn20, all prepared with the same current density, an interesting relation is visible. All the described variations have shown similar anodic peak potential during UOR in 1 M KOH + 0.15 M urea solution, ranging from 1.7 to 1.74 V_vs RHE_. Observed peak current densities vary more strongly, with the lowest *i*_pA_ of 3.9 mA cm^−2^ observed for NiCu catalyst [22], increasing with each addition to 5.9 mA cm^−2^ after the addition of graphene oxide [22], to 10 mA cm^−2^ after the addition of zinc, and to 12 mA cm^−2^ after the addition of iron [30]. Observed dependence shows that the presence of another metallic element is more beneficial for the UOR activity of the NiCu-based system prepared by electrodeposition than the presence of nanoscale carbon particles, such as graphene oxide. This relation shows that the electronic effect is influencing the catalyst activity more strongly than the increase in material conductivity and surface development caused by the preparation of the composite material.

Although the maximum current densities achieved by NiCuZn20 are lower than those reported for advanced NiO structures or graphene-based catalysts, which often exhibit exceptionally high current values, it should be noted that such materials are typically produced using hydrothermal or multistep synthesis procedures. These approaches require extended synthesis times and stringent control of processing conditions.

The presence of nanoscale carbon particles of high conductivity provides a conducting support for catalyst particles and thus increases the number of anchoring sites, preventing the particles from aggregating. Thanks to this property, the usage of graphene as a supporting material allows better utilization of the used material, which leads to higher activity of the final catalyst [28,33,63]. Additionally, the usage of smaller particles results in higher activity of the final material due to its larger electrochemically active area, which is crucial for surface processes, such as an electrocatalyst [64].

Both conclusions suggest that further increases in NiCuZn catalyst activity might be obtained by changing the electrosynthesis process conditions to obtain smaller particles, which should be further investigated in further studies.

**Figure 7:**
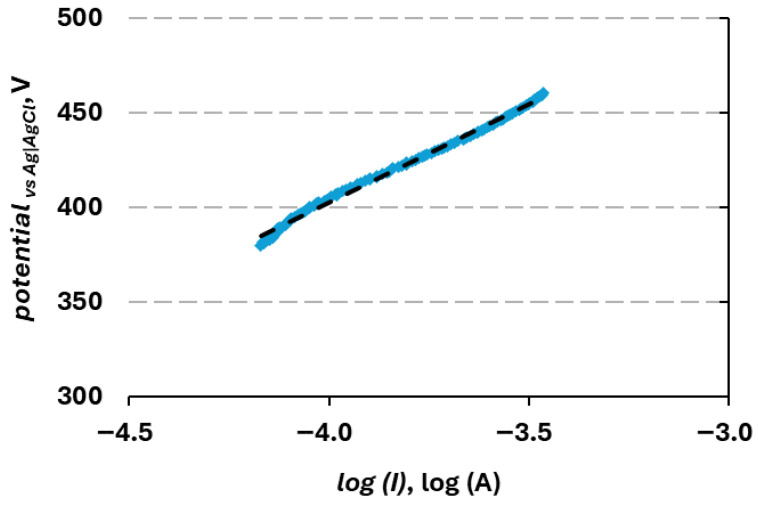
Representative Tafel plot registered for NiCuZn20 sample in 1 M KOH + 0.15 M urea solution. Dashed line corresponds to a trend line calculated on the base of obtained results.

Urea oxidation reaction kinetics can be characterized by a Tafel plot, showing how big a potential change is necessary for the increase in current density by a decade (Figure 7). For NiCuZn20, the average Tafel slope value was 117.4 ± 15.9 mV dec^−1^. The obtained value lies within the already reported in the literature for similar materials, such as 118.12 mV/dec for Zn-NiCo_2_S_4_ [65] and 112.5 mV dec^−1^ for Co/Zn-OH@NF [66]. However, observed values are higher than already reported for composite equivalents like NiCuGO and the binary electrode NiCu, which were characterized with the values of 36 and 82, respectively [22], which might indicate that the presence of zinc is negatively influencing the UOR kinetics. Higher activity observed for NiCuZn, despite a higher Tafel slope, suggests that the limiting factor for UOR on this branch of materials is the reactant diffusion.

From a practical perspective, therefore, the moderate current densities obtained for NiCuZn20, combined with the simplicity and rapidity of the electrodeposition process, constitute a clear advantage.

### 3.3. Stability Examination

#### 3.3.1. Multiscan Test

**Figure 8:**
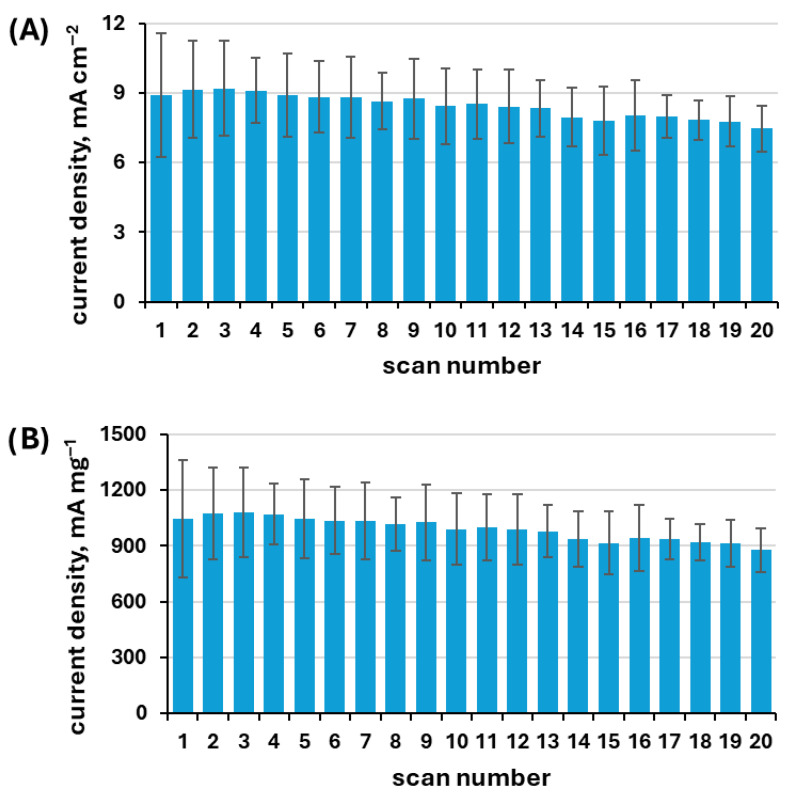
Anodic peak current density (**A**) and corresponding specific activity (**B**) recorded for the NiCuZn20 sample over 20 consecutive cyclic voltammetry (CV) scans in 1 M KOH containing 0.15 M urea.

Tests were conducted to evaluate the stability of the system under repeated use of the catalyst. The obtained results of the multiscan stability test are presented in Figure 8.

The anodic peak current density (A) and specific activity (B) of the NiCuZn20 catalyst were monitored over 20 successive CV scans in 1 M KOH containing 0.15 M urea. The anodic peak current density values fluctuated slightly between approximately 7.4 and 9.3 mA cm^−2^, while the specific activity ranged from around 870 to 1080 mA mg^−1^. No significant downward trend was observed and the variations remained within a narrow range, indicating high catalytic stability and good reproducibility across multiple measurements.

#### 3.3.2. Chronoamperometry

**Figure 9:**
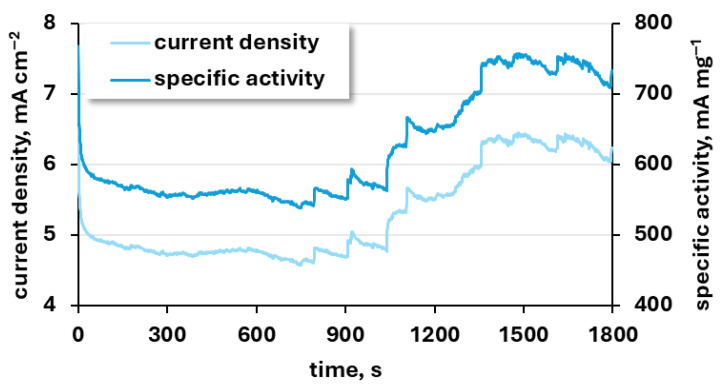
Anodic peak current density and corresponding specific activity recorded for the NiCuZn20 sample during 1800 s of polarization at the anodic peak potential in 1 M KOH solution containing 0.15 M urea.

The chronoamperometric study was performed to assess the electrochemical stability of the NiCuZn powders under continuous operation during urea oxidation (Figure 9). This measurement allows evaluation of the catalyst’s durability and activity retention over time under constant anodic polarization.

The anodic current density remained relatively stable during the initial 1100 s of polarization, fluctuating slightly around 5.0 mA cm^−2^ (570 mA mg^−1^). Notably, in the latter stage of the measurement (1100–1800 s), a gradual increase in current density was observed, eventually reaching approximately 6.5 mA cm^−2^ (740 mA mg^−1^). The observed peak may be attributed to the increased availability of active sites following product desorption. In the literature, a more linear trend has typically been reported, with the current density remaining relatively constant over time. However, it should be noted that the overall current density reported for other catalysts was higher [67,68].

**Figure 10:**
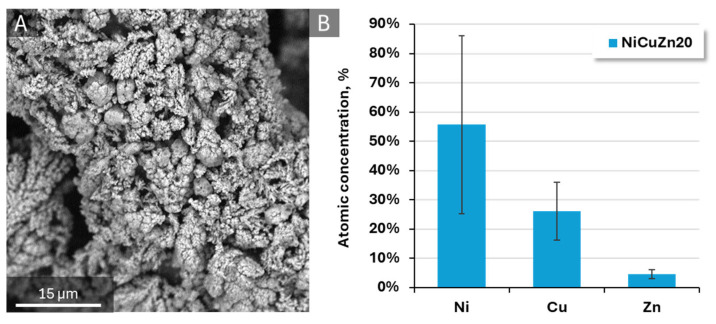
SEM image of the working electrode after the chronoamperometric stability test (**A**) together with the corresponding EDX analysis (**B**), illustrating the morphological and compositional changes induced by prolonged electrochemical operation in 1 M KOH with 0.15 M urea.

To examine the influence of the electrochemical process on the investigated catalyst, EDX was also conducted after the electrochemical process. Figure 10 shows the surface morphology of the NiCuZn20 working electrode after chronoamperometric testing, along with the corresponding EDX analysis of elemental composition. The SEM image (Figure 10A) reveals a rough and porous structure, with interconnected agglomerates forming a three-dimensional network. The EDX results (Figure 10B) show that NiCuZn20 contains about 55 ± 30% of nickel, 25 ± 10% of copper, and 4 ± 2% of zinc (atomic %) after chronoamperometry testing. The copper and zinc contents decreased compared to the initially measured values for the unused catalyst (Figure 2). In particular, the copper content decreased by approximately ten percentage points. Zinc concentration went from 16 ± 2% to 4 ± 2% but was still present in the material. This behaviour may be associated with partial zinc etching observed during the stability tests—both the chronoamperometric studies and the multiscan tests. After etching of zinc from the material surface, a higher amount of UOR active centres might be in contact with the solution, leading to higher observed current. More porous material might also be characterized by higher activity due to easier diffusion of reactants. Additionally, partial Zn etching might result in a more beneficial Ni:Cu:Zn ratio of the material and thus, improve the material activity by increasing its ability to form NiOOH and Ni^4+^ species, which are active centres for urea oxidation [69,70].

Electrochemical stability represents an important strength of the powders examined in this work. Multiscan measurements demonstrate stable anodic peak current density and specific activity over repeated cycles. Furthermore, NiCuZn20 maintains a steady current response during anodic polarization. The gradual change in current density observed during chronoamperometric testing can be attributed to partial zinc leaching, which increases the electrochemically active surface area and improves the accessibility of nickel active sites responsible for urea oxidation. Comparable behaviour has been reported for other multicomponent nickel-based anodes.

Of the two powders studied, NiCuZn20 exhibits superior electrochemical performance compared to NiCuZn15. This enhancement is likely related to higher nickel contents and its more homogeneous morphology and finer grain structure, which promote a higher density of accessible active sites. Moreover, the distinct responses of the two materials to changes in KOH concentration further confirm that the catalyst’s composition and microstructure play a crucial role in the electrochemical processes.

The catalyst preparation process itself also offers several advantages, ranging from a straightforward methodology and the absence of sophisticated equipment requirements to high versatility. As proven above, a single electrodeposition bath enables the fabrication of materials with diverse characteristics by adjusting the synthesis parameters.

In conclusion, although NiCuZn does not deliver the highest current densities reported for urea oxidation anodes, its balanced combination of catalytic activity, electrochemical stability, and facile synthesis renders it a competitive anode material for direct urea fuel cell applications. These findings demonstrate that electrodeposited NiCuZn systems represent a promising and cost-effective alternative to more complex nickel-based catalysts. The results obtained are encouraging and highlight the need for continued research in this area.

## 4. Conclusions

In this study, ternary NiCuZn powders were successfully synthesized via a simple and rapid electrodeposition method, providing a cost-effective route for the preparation of anode materials for urea oxidation. SEM and EDX analyses confirmed the formation of granular, coral-like deposits, with the NiCuZn20 sample exhibiting a more homogeneous morphology and finer grain structure compared to NiCuZn15. The applied current density was shown to significantly influence both the morphology and elemental composition of the obtained materials. Electrochemical measurements demonstrated that the synthesis conditions strongly affect the catalytic performance of the materials. All samples exhibited the characteristic Ni(II)/Ni(III) redox transition in alkaline media, while NiCuZn20 displayed superior activity in 1 M KOH solution and therefore selected for further investigation. A pronounced increase in anodic current upon urea addition confirmed its catalytic functionality. Moreover, the catalyst showed stable performance during multicycle cyclic voltammetry and chronoamperometric measurements. Post-stability analysis indicated partial zinc leaching, which may influence active site accessibility and reaction pathways. The maximum specific activity achieved under the applied conditions reached 740 mA mg^−1^.

Beyond the immediate electrochemical performance, this work highlights broader scientific and practical implications. In particular, it demonstrates that electrodeposited NiCuZn systems represent a promising class of cost-effective and compositionally tunable electrocatalysts. The ability to tailor material properties through electrochemical parameters, such as current density, offers significant flexibility in catalyst design. Furthermore, the observed compositional changes during operation emphasize the importance of considering catalyst surface evolution when evaluating activity and stability in urea oxidation systems.

Overall, although the NiCuZn catalysts do not exhibit the highest current densities reported in the literature, their balanced combination of satisfactory catalytic activity, good electrochemical stability, and facile scalable synthesis renders them competitive anode materials for direct urea fuel cell applications. The results obtained highlight the potential of simplified electrodeposition routes as viable alternatives to more complex, multistep synthesis methods. Future research should focus on a deeper understanding of the reaction mechanism through detailed kinetic studies and extended long-term stability testing. Advanced post-reaction characterization techniques, such as XPS, ICP-OES, XRD, and Raman spectroscopy, will be essential for elucidating changes in surface composition, oxidation states, and phase structure. Additionally, further compositional engineering, including controlled doping with other transition metals, represents a promising strategy for enhancing both catalytic activity and durability.

## Figures and Tables

**Table 1:** Composition of the electrolytic bath used in the experiments.

Ingredient	Concentration [M]	pH
NiSO_4_	0.375	9
CuSO_4_	0.125	
Sodium citrate	0.2	
ZnSO_4_	0.1	

**Table 2:** Average peak anodic peak current densities registered for the examined samples in KOH solutions of various concentrations.

[KOH], M	NiCuZn15		NiCuZn20	
	*i*_pA_, mA mg^−1^	*i*_pA_, mA cm^−2^	*i*_pA_, mA mg^−1^	*i*_pA_, mA cm^−2^
0.5	158.1 ± 6.4	1.34 ± 0.05	155.6 ± 49.0	1.32 ± 0.42
1	85.3 ± 3.0	0.73 ± 0.03	126.3 ± 5.3	1.07 ± 0.04
2	74.2 ± 1.3	0.63 ± 0.01	344.8 ± 181.4	2.93 ± 1.54

**Table 3:** Literature comparison of selected nickel and nickel-based materials employed as anodes for the electrochemical oxidation of urea in alkaline media.

Material	Solution	Onset Potential, *E* _vs RHE_, V	Peak Potential, *E* _vs RHE_, V	Peak Current Density [mA cm^−2^]	Reference
NiCuZn20	0.15 M urea/1 M KOH	1.42	1.72	10.0	-
Ni-Cu-Fe	0.15 M urea/1 M KOH		1.70	12.0	[30]
Ni-Zn-Co	0.33 M urea/5 M KOH	1.27	1.44	24.0	[18]
NiO	0.4 M urea/6 M KOH	1.26	1.37	20.0	[5]
NiO/Gr	0.3 M urea/0.5 M NaOH	1.40	1.73	30.9	[34]
Ni/Pd-C	0.3 M urea/2 M KOH	1.37	1.47	63.0	[6]
NiFe-LDH	0.33 M urea/1 M KOH		1.30	10.0	[19]
Ni/Gr	0.33 M urea/1 M KOH	1.39	1.92	81.7	[9]
Ni@GO	0.33 M urea/1 M KOH	1.37	1.56	17.1	[28]
Ultrafine NiO nanoparticles	0.25 M urea/1 M KOH		1.42	216.1	[37]
NiCuGO20	0.15 M urea/1 M KOH		1.74	5.9	[22]
NiCu20	0.15 M urea/1 M KOH		1.73	3.9	[22]
Ni@C	0.1 M urea/0.1 M KOH	1.41	1.62	40.0	[17]
NiO@C	0.1 M urea/0.1 M KOH	1.35	1.67	25.0	[17]
Ni Cu/ZnO@ MWCNT	0.07 M urea/0.4 M KOH		1.40	30.0	[33]
NiFeTiS	0.33 M urea/1 M KOH		2.38	100.0	[41]
Ni/C		1.33			[62]

For easier comparison, the reported values of onset and peak potentials in reference to silver chloride (Ag|AgCl), mercury oxide (Hg|HgO), saturated calomel (SCE), and standard hydrogen electrode (SHE) have been converted *versus* the reversible hydrogen electrode (RHE) according to: *E*_RHE_ = *E*_Ag|AgCl_ + 0.197 + 0.059 pH, *E*_RHE_ = *E*_Hg|HgO_ + 0.098 + 0.059 pH, and *E*_RHE_ = *E*_SCE_ + 0.241 + 0.059 pH.

## Data Availability

The original contributions presented in this study are included in the article. Further inquiries can be directed to the corresponding author.

## References

[1] Farghali M., Osman A.I., Mohamed I.M.A., Chen Z., Chen L., Ihara I., Yap P.S., Rooney D.W. (2023). Strategies to save energy in the context of the energy crisis: A review. Environ. Chem. Lett..

[2] Guaitolini S.V.M., Fardin J.F. (2018). Fuel Cells: History, Principles of Operation, Main Features, and Applications. Adv. Renew. Energ. Power Technol..

[3] Vakulchuk R., Overland I., Scholten D. (2020). Renewable energy and geopolitics: A review. Renew. Sustain. Energy Rev..

[4] Moriarty P., Honnery D. (2012). What is the global potential for renewable energy? *Renew*. Sustain. Energy Rev..

[5] Alex C., Shukla G., John N.S. (2021). Introduction of surface defects in NiO with effective removal of adsorbed catalyst poisons for improved electrochemical urea oxidation. Electrochim. Acta.

[6] Mohamed I.M.A., Kanagaraj P., Yasin A.S., Iqbal W., Liu C. (2020). Electrochemical impedance investigation of urea oxidation in alkaline media based on electrospun nanofibers towards the technology of direct-urea fuel cells. J. Alloys Compd..

[7] Gnana Kumar G., Farithkhan A., Manthiram A. (2020). Direct Urea Fuel Cells: Recent Progress and Critical Challenges of Urea Oxidation Electrocatalysis. Adv. Energy Sustain. Res..

[8] Yu X., Williams C.T. (2023). Recent applications of nickel and nickel-based bimetallic catalysts for hydrodeoxygenation of biomass-derived oxygenates to fuels. Catal. Sci. Technol..

[9] Yousef A., El-Newehy M.H., Al-Deyab S.S., Barakat N.A.M. (2017). Facile synthesis of Ni-decorated multi-layers graphene sheets as effective anode for direct urea fuel cells. Arab. J. Chem..

[10] Ahmadi P., Torabi S.H., Afsaneh H., Sadegheih Y., Ganjehsarabi H., Ashjaee M. (2020). The effects of driving patterns and PEM fuel cell degradation on the lifecycle assessment of hydrogen fuel cell vehicles. Int. J. Hydrogen Energy.

[11] King R.L., Botte G.G. (2011). Hydrogen production via urea electrolysis using a gel electrolyte. J. Power Sources.

[12] Shekhawat A., Samanta R., Panigrahy S., Barman S. (2023). Electrocatalytic Oxidation of Urea and Ethanol on Two-Dimensional Amorphous Nickel Oxide Encapsulated on N-Doped Carbon Nanosheets. ACS Appl. Energy Mater..

[13] Urbańczyk E., Wala M., Blacha-Grzechnik A., Stolarczyk A., Maciej A., Simka W. (2021). Electrocatalytic methanol oxidation using Ni–Co–graphene composite electrodes. Int. J. Hydrogen Energy.

[14] Mansor M., Timmiati S.N., Lim K.L., Wong W.Y., Kamarudin S.K., Nazirah Kamarudin N.H. (2019). Recent progress of anode catalysts and their support materials for methanol electrooxidation reaction. Int. J. Hydrogen Energy.

[15] Zakaria Z., Kamarudin S.K., Wahid K.A.A. (2023). Polymer electrolyte membrane modification in direct ethanol fuel cells: An update. J. Appl. Polym. Sci..

[16] Baruah S., Kumar A., Peela N.R. (2025). Advancing paper-based microfluidic ethanol fuel cells with gel-assisted dual electrolytes: A step towards scalable power solutions. Energy Convers. Manag..

[17] Tran T.Q.N., Park B.J., Yun W.H., Duong T.N., Yoon H.H. (2020). Metal–organic framework–derived Ni@C and NiO@C as anode catalysts for urea fuel cells. Sci. Rep..

[18] Yan W., Wang D., Botte G.G. (2012). Electrochemical decomposition of urea with Ni-based catalysts. Appl. Catal. B Environ..

[19] Liu G., Xie C., Zhang Y., Du Y., Wang J., Lin J., Bai J., Li J., Zhou C., Zhou T. (2024). Synergistic etching of nickel foam by Fe3+ and Cl− ions to synthesize nickel-iron-layered double hydroxide nanolayers with abundant oxygen vacancies for superior urea oxidation. J. Colloid Interface Sci..

[20] Cai M., Zhu Q., Wang X., Shao Z., Yao L., Zeng H., Wu X., Chen J., Huang K., Feng S. (2023). Formation and Stabilization of NiOOH by Introducing α-FeOOH in LDH: Composite Electrocatalyst for Oxygen Evolution and Urea Oxidation Reactions. Adv. Mater..

[21] Yang K., Hao L., Hou Y., Zhang J., Yang J.H. (2024). Summary and application of Ni-based catalysts for electrocatalytic urea oxidation. Int. J. Hydrogen Energy.

[22] Wala M., Blacha–Grzechnik A., Stolarczyk A., Bajkacz S., Dydo P., Simka W. (2023). Unexpected electrochemical oxidation of urea on a new NiCuGO composite catalyst. Int. J. Hydrogen Energy.

[23] Goel R., Jha R., Ravikant C. (2020). Investigating the structural, electrochemical, and optical properties of p-type spherical nickel oxide (NiO) nanoparticles. J. Phys. Chem. Solids.

[24] Tariq I., Iqbal W., Haider A., Ma M. (2025). Recent advances in carbon nanotube-supported non-noble metal electrocatalysts for urea oxidation reaction. Int. J. Hydrogen Energy.

[25] Ma Y., Ma C., Wang Y., Wang K. (2022). Advanced Nickel-Based Catalysts for Urea Oxidation Reaction: Challenges and Developments. Catalysts.

[26] Tatarchuk S.W., Medvedev J.J., Li F., Tobolovskaya Y., Klinkova A. (2022). Nickel-Catalyzed Urea Electrolysis: From Nitrite and Cyanate as Major Products to Nitrogen Evolution. Angew. Chem..

[27] Li S.-M., Zhang H.-R., Liu J.-H. (2007). Corrosion behavior of aluminum alloy 2024-T3 by 8-hydroxy-quinoline and its derivative in 3.5% chloride solution. Trans. Nonferrous Met. Soc. China.

[28] Munde A.V., Mulik B.B., Chavan P.P., Sathe B.R. (2020). Enhanced electrocatalytic activity towards urea oxidation on Ni nanoparticle decorated graphene oxide nanocomposite. Electrochim. Acta.

[29] Guo F., Ye K., Du M., Huang X., Cheng K., Wang G., Cao D. (2016). Electrochemical impedance analysis of urea electro-oxidation mechanism on nickel catalyst in alkaline medium. Electrochim. Acta.

[30] Wala-Kapica M., Gąsior A., Maciej A., Smykała S., Kazek-Kęsik A., Baghayeri M., Simka W. (2024). One-Pot Fast Electrochemical Synthesis of Ternary Ni-Cu-Fe Particles for Improved Urea Oxidation. Energies.

[31] Li J., Wang S., Chang J., Feng L. (2022). A review of Ni based powder catalyst for urea oxidation in assisting water splitting reaction. Adv. Powder Mater..

[32] Guo F., Cao D., Du M., Ye K., Wang G., Zhang W., Gao Y., Cheng K. (2016). Enhancement of direct urea-hydrogen peroxide fuel cell performance by three-dimensional porous nickel-cobalt anode. J. Power Sources.

[33] Basumatary P., Konwar D., Yoon Y.S. (2018). A novel Ni–Cu/ZnO@MWCNT anode employed in urea fuel cell to attain superior performances. Electrochim. Acta.

[34] Abdel Hameed R.M., Medany S.S. (2017). NiO nanoparticles on graphene nanosheets at different calcination temperatures as effective electrocatalysts for urea electro-oxidation in alkaline medium. J. Colloid Interface Sci..

[35] Wang D., Yan W., Vijapur S.H., Botte G.G. (2013). Electrochemically reduced graphene oxide–nickel nanocomposites for urea electrolysis. Electrochim. Acta.

[36] Ye K., Zhang D., Guo F., Cheng K., Wang G., Cao D. (2015). Highly porous nickel@carbon sponge as a novel type of three-dimensional anode with low cost for high catalytic performance of urea electro-oxidation in alkaline medium. J. Power Sources.

[37] Abd El-Lateef H.M., Almulhim N.F., Alaulamie A.A., Saleh M.M., Mohamed I.M.A. (2020). Design of ultrafine nickel oxide nanostructured material for enhanced electrocatalytic oxidation of urea: Physicochemical and electrochemical analyses. Colloids Surf. A Physicochem. Eng. Asp..

[38] Chakrabarty S., Offen-Polak I., Burshtein T.Y., Farber E.M., Kornblum L., Eisenberg D. (2021). Urea oxidation electrocatalysis on nickel hydroxide: The role of disorder. J. Solid State Electrochem..

[39] Xiao M., Tian Y., Yan Y., Feng K., Miao Y. (2015). Electrodeposition of Ni(OH)2/NiOOH in the Presence of Urea for the Improved Oxygen Evolution. Electrochim. Acta.

[40] Hakami W., Danish E.Y., Khan A.N., Khudaysh A., Aslam M., Soomro M.T. (2025). An innovative electrochemical approach for selective and sensitive urea sensing using NiNPs modified GCE. Sci. Rep..

[41] Nur Indah Sari F., Ke M.T., Huang Y.J., Zheng T.M., Su Y.H., Ting J.M. (2024). NiFe sulfide electronic structure modulation via metal doping towards enhanced urea oxidation reaction performance. Appl. Surf. Sci..

[42] Wickramaarachchi K., Minakshi M. (2022). Status on electrodeposited manganese dioxide and biowaste carbon for hybrid capacitors: The case of high-quality oxide composites, mechanisms, and prospects. J. Energy Storage.

[43] Plowman B.J., Jones L.A., Bhargava S.K. (2015). Building with bubbles: The formation of high surface area honeycomb-like films via hydrogen bubble templated electrodeposition. Chem. Commun..

[44] Li Y., Jia W.Z., Song Y.Y., Xia X.H. (2007). Superhydrophobicity of 3D Porous Copper Films Prepared Using the Hydrogen Bubble Dynamic Template. Chem. Mater..

[45] Zhang Z., Xie M., Liu Z., Lu Y., Zhang S., Liu M., Liu K., Cheng T., Gao C. (2021). Ultrathin Pt-Cu-Ni Ternary Alloy Nanowires with Multimetallic Interplay for Boosted Methanol Oxidation Activity. ACS Appl. Energy Mater..

[46] Wang C., Du X., Zhang X. (2022). Controlled synthesis of Fe doped NiCoM (M=O, P, S and Se) as robust electrocatalyst for urea electrolysis. J. Alloys Compd..

[47] Li Q., Zhang W., Shen J., Zhang X., Liu Z., Liu J. (2022). Trimetallic nanoplate arrays of Ni-Fe-Mo sulfide on FeNi3 foam: A highly efficient and bifunctional electrocatalyst for overall water splitting. J. Alloys Compd..

[48] Wala M., Szewczyk M., Leśniak–Ziółkowska K., Kazek–Kęsik A., Simka W. (2022). Preparation of NiCuGO composite and investigation of its electrocatalytic properties in methanol oxidation. Electrochim. Acta.

[49] Wu M.S., Chen F.Y., Lai Y.H., Sie Y.J. (2017). Electrocatalytic oxidation of urea in alkaline solution using nickel/nickel oxide nanoparticles derived from nickel-organic framework. Electrochim. Acta.

[50] Luo Y., Zhou H., Tong Y. (2026). Advances in Nanostructured Catalysts for Urea-Assisted Water Splitting and Zn-Urea Batteries. ChemSusChem.

[51] Chu S., Chen W., Chen G., Huang J., Zhang R., Song C., Wang X., Li C., Ostrikov K. (2019). Holey Ni-Cu phosphide nanosheets as a highly efficient and stable electrocatalyst for hydrogen evolution. Appl. Catal. B Environ..

[52] Zhang H.M., Wang Y.F., Kwok Y.H., Wu Z.C., Xia D.H., Leung D.Y.C. (2018). A Direct Ammonia Microfluidic Fuel Cell using NiCu Nanoparticles Supported on Carbon Nanotubes as an Electrocatalyst. ChemSusChem.

[53] Yuan L.S., Zheng Y.X., Jia M.L., Zhang S.J., Wang X.L., Peng C. (2015). Nanoporous nickel-copper-phosphorus amorphous alloy film for methanol electro-oxidation in alkaline medium. Electrochim. Acta.

[54] Noor T., Pervaiz S., Iqbal N., Nasir H., Zaman N., Sharif M., Pervaiz E. (2020). Nanocomposites of NiO/CuO based MOF with rGO: An efficient and robust electrocatalyst for methanol oxidation reaction in DMFC. Nanomaterials.

[55] Wittman R.M., Sacci R.L., Zawodzinski T.A. (2025). Alkaline Zinc Passivation Mechanism is Controlled by Hydroxide Concentration. J. Electrochem. Soc..

[56] Hampson N.A., Shawt P.E., Taylor R. (1969). Anodic Behaviour of Zinc in Potassium Hydroxide Solution: II. Horizontal Anodes in Electrolytes Containing Zn(II). Br. Corros. J..

[57] Vedharathinam V., Botte G.G. (2012). Understanding the electro-catalytic oxidation mechanism of urea on nickel electrodes in alkaline medium. Electrochim. Acta.

[58] Hopsort G., Carmo D.P.D., Latapie L., Loubière K., Serrano K.G., Tzedakis T. (2023). Progress toward a better understanding of the urea oxidation by electromediation of Ni(III)/Ni(II) system in alkaline media. Electrochim. Acta.

[59] Schwab B., Ruh A., Manthey J., Drosik M.Z. (2015). Ullmann’s Encycl. Ind. Chem..

[60] Merten H. (1983). Zinc Alloys. Mold-Mak. Handb. Plast. Eng..

[61] Ayyanusamy P., Swathi Tharani D., Alphonse R., Minakshi M., Sivasubramanian R. (2024). Synthesis of Amorphous Nickel-Cobalt Hydroxides for Ni−Zn Batteries. Chem. A Eur. J..

[62] Wang L., Ren L., Wang X., Feng X., Zhou J., Wang B. (2018). Multivariate MOF-Templated Pomegranate-Like Ni/C as Efficient Bifunctional Electrocatalyst for Hydrogen Evolution and Urea Oxidation. ACS Appl. Mater. Interfaces.

[63] Liu M., Zhang R., Chen W. (2014). Graphene-Supported Nanoelectrocatalysts for Fuel Cells: Synthesis, Properties, and Applications. Chem. Rev..

[64] Li J., Wang S., Sun S., Wu X., Zhang B., Feng L. (2022). A review of hetero-structured Ni-based active catalysts for urea electrolysis. J. Mater. Chem. A.

[65] Wang Y., Chen N., Du X., Han X., Zhang X. (2022). Transition metal atoms M (M = Mn, Fe, Cu, Zn) doped nickel-cobalt sulfides on the Ni foam for efficient oxygen evolution reaction and urea oxidation reaction. J. Alloys Compd..

[66] Zhang R., Wang Y., Gao X., Duan D., Wang J., Liu S. (2024). Electrodeposited zinc cobalt bimetallic phosphate as a bifunctional catalyst for hydrogen evolution and urea oxidation. J. Alloys Compd..

[67] Clark R., Moore A., MacInnis M., Bertin E. (2021). Investigation of urea oxidation as a potential anode reaction during CO_2_ electrolysis. J. Appl. Electrochem..

[68] Carpenter K., Stuve E.M. (2021). Electrooxidation of urea and creatinine on nickel foam-based electrocatalysts. J. Appl. Electrochem..

[69] Zhang H.J., Chen Z.Q., Ye X.T., Xiao K., Liu Z.Q. (2025). Electron delocalized ni active sites in spinel catalysts enable efficient urea oxidation Angew. Chem. Int. Ed..

[70] Bender M.T., Lam Y.C., Hammes-Schiffer S., Choi K.S. (2020). Unraveling Two Pathways for Electrochemical Alcohol and Aldehyde Oxidation on NiOOH. J. Am. Chem. Soc..

